# LPG stove and fuel intervention among pregnant women reduce fine particle air pollution exposures in three countries: Pilot results from the HAPIN trial^☆^

**DOI:** 10.1016/j.envpol.2021.118198

**Published:** 2021-12-15

**Authors:** Jiawen Liao, Miles A. Kirby, Ajay Pillarisetti, Ricardo Piedrahita, Kalpana Balakrishnan, Sankar Sambandam, Krishnendu Mukhopadhyay, Wenlu Ye, Ghislaine Rosa, Fiona Majorin, Ephrem Dusabimana, Florien Ndagijimana, John P. McCracken, Erick Mollinedo, Oscar de Leon, Anaité Díaz-Artiga, Lisa M. Thompson, Katherine A. Kearns, Luke Naeher, Joshua Rosenthal, Maggie L. Clark, Kyle Steenland, Lance A. Waller, William Checkley, Jennifer L. Peel, Thomas Clasen, Michael Johnson, on behalf of the HAPIN Investigators, Vigneswari Aravindalochanan, Gloriose Bankundiye, Dana Boyd Barr, Alejandra Bussalleu, Eduardo Canuz, Adly Castañaza, Yunyun Chen, Marilú Chiang, Rachel Craik, Victor G. Davila-Roman, Lisa de las Fuentes, Lisa Elon, Juan Gabriel Espinoza, Sarada Garg, Sarah Hamid, Stella Hartinger, Steven A. Harvey, Mayari Hengstermann, Ian Hennessee, Phabiola M. Herrera, Shakir Hossen, Penelope P. Howards, Lindsay Jaacks, Shirin Jabbarzadeh, Pattie Lenzen, Amy E. Lovvorn, Jane Mbabazi, Eric McCollum, Rachel Meyers, Lawrence Moulton, Alexie Mukeshimana, Bernard Mutariyani, Durairaj Natesan, Azhar Nizam, Jean de Dieu Ntivuguruzwa, Aris Papageorghiou, Naveen Puttaswamy, Elisa Puzzolo, Ashlinn Quinn, Karthikeyan Dharmapuri Rajamani, Usha Ramakrishnan, Rengaraj Ramasami, Alexander Ramirez, P. Barry Ryan, Sudhakar Saidam, Jeremy A. Sarnat, Suzanne Simkovich, Sheela S. Sinharoy, Kirk R. Smith, Damien Swearing, Gurusamy Thangavel, Ashley Toenjes, Viviane Valdes, Kendra N. Williams, Wenlu Ye, Bonnie N. Young

**Affiliations:** a Gangarosa Department of Environmental Health, Emory University Rollins School of Public Health, Atlanta, GA, USA; b Department of Population and Public Health Sciences, Keck School of Medicine of the University of Southern California, Los Angeles, CA, USA; c Department of Global Health and Population, Harvard T.H. Chan School of Public Health, Boston, MA, USA; d School of Public Health, University of California, Berkeley, CA, USA; e Berkeley Air Monitoring Group, Berkeley, CA, USA; f SRU-ICMR Center for Advanced Research on Air Quality, Climate and Health, Sri Ramachandra Institute of Higher Education and Research, Chennai, India; g London School of Hygiene and Tropical Medicine, London, UK; h Eagle Research Center, Kigali, Rwanda; i Center for Health Studies, Universidad del Valle De Guatemala, Guatemala City, Guatemala; j College of Public Health, University of Georgia, Athens, GA, USA; k Nell Hodgson Woodruff School of Nursing, Emory University, Atlanta, GA, USA; l Fogarty International Center, National Institutes of Health, Bethesda, MD, USA; m Department of Environmental and Radiological Health Sciences, Colorado State University, Fort Collins, CO, USA; n Division of Pulmonary and Critical Care, School of Medicine, Johns Hopkins University, Baltimore, MD, USA; o Center for Non-Communicable Disease Research and Training, School of Medicine, Johns Hopkins University, Baltimore, MD, USA

**Keywords:** Household air pollution, Cookstove, Clean cooking fuel, Intervention, PM_2.5_, Personal exposure

## Abstract

The Household Air Pollution Intervention Network trial is a multi-country study on the effects of a liquefied petroleum gas (LPG) stove and fuel distribution intervention on women's and children's health. There is limited data on exposure reductions achieved by switching from solid to clean cooking fuels in rural settings across multiple countries. As formative research in 2017, we recruited pregnant women and characterized the impact of the intervention on personal exposures and kitchen levels of fine particulate matter (PM_2.5_) in Guatemala, India, and Rwanda. Forty pregnant women were enrolled in each site. We measured cooking area concentrations of and personal exposures to PM_2.5_ for 24 or 48 h using gravimetric-based PM_2.5_ samplers at baseline and two follow-ups over two months after delivery of an LPG cookstove and free fuel supply. Mixed models were used to estimate PM_2.5_ reductions. Median kitchen PM_2.5_ concentrations were 296 μg/m^3^ at baseline (interquartile range, IQR: 158–507), 24 μg/m^3^ at first follow-up (IQR: 18–37), and 23 μg/m^3^ at second follow-up (IQR: 14–37). Median personal exposures to PM_2.5_ were 134 μg/m^3^ at baseline (IQR: 71–224), 35 μg/m^3^ at first follow-up (IQR: 23–51), and 32 μg/m^3^ at second follow-up (IQR: 23–47). Overall, the LPG intervention was associated with a 92% (95% confidence interval (CI): 90–94%) reduction in kitchen PM_2.5_ concentrations and a 74% (95% CI: 70–79%) reduction in personal PM_2.5_ exposures. Results were similar for each site.

**Conclusions:**

The intervention was associated with substantial reductions in kitchen and personal PM_2.5_ overall and in all sites. Results suggest LPG interventions in these rural settings may lower exposures to the WHO annual interim target-1 of 35 μg/m^3^. The range of exposure contrasts falls on steep sections of estimated exposure-response curves for birthweight, blood pressure, and acute lower respiratory infections, implying potentially important health benefits when transitioning from solid fuels to LPG.

## Highlights

• There was high uptake of an LPG stove and fuel intervention in 3 rural settings.
• The LPG intervention was associated with a 92% reduction in kitchen PM_2.5_ levels.
• The intervention was associated with a 74% reduction in personal exposure to PM_2.5_
• Maternal PM_2.5_ exposure was reduced to the WHO annual interim target-1 of 35 μg/m^3^.
• Results from 3 countries indicate switching to clean fuel could improve health.

## Teaser

Capsule summary: LPG stove and fuel intervention can reduce kitchen area and personal PM_2.5_ exposure levels in a pilot study of clean cookstove intervention trial. This strongly suggests switching to clean cook can improve health.

## 1 Introduction

Household air pollution due to use of solid fuels such as wood and charcoal for cooking, heating and lighting is a major risk factor for morbidity and premature mortality (GBD, 2017 Risk Factor Collaborators, 2018). Over 3 billion households rely on solid fuels for cooking, primarily in low-income settings (Bonjour et al., 2013; Health Effects Institute, 2019; WHO, 2016). To date, solid fuel cooking interventions have focused on improved combustion efficiency or ventilation and have largely failed to demonstrate substantial exposure reductions or health benefits (Baumgartner et al., 2019; Kirby et al., 2019; Mortimer et al., 2020, 2016; Pope et al., 2017; Quansah et al., 2017).

Thus, clean cooking fuels, such as electricity, ethanol, biogas, and liquefied petroleum gas (LPG), are widely considered necessary for obtaining exposure reductions and accompanying health and climate benefits (Grieshop et al., 2011). Scale-up of these clean technologies would likely contribute to reaching several Sustainable Development Goals (Rosenthal et al., 2018), including Good health and well-being [SDG 3], Gender equality [SDG 5], Affordable and clean energy [SDG 7], and Climate action [SDG13]. LPG, in particular, is amenable to large-scale dissemination and uptake, and clean cooking programs focusing on LPG are being scaled up worldwide (Gould and Urpelainen, 2018; Quinn et al., 2018).

LPG stoves in lab settings have been shown to reach or surpass the WHO emission rate target guidance for PM_2.5_ emissions (0.23 mg/min) (Johnson et al., 2014; Shen et al., 2018; WHO, 2014) and perform similarly in real-world field conditions (Johnson et al., 2019). However, relatively few studies have assessed exposure reductions that can be obtained by transitioning to cleaner fuels like LPG, particularly in rural settings, where access to these fuels is limited (Pillarisetti et al., 2019b; Pope et al., 2017). The potential for clean cooking fuels to improve health likely depends primarily on high adherence (Johnson and Chiang, 2015; Snider et al., 2018) and on the ability to reduce exposure to levels below or near the WHO annual PM_2.5_ Interim Target-1 (IT-1) of 35 μg/m^3^ (Bruce et al., 2015; Burnett et al., 2014; WHO, 2014). While it is believed that switching fuel types can result in these reductions in personal exposure and yield health benefits, there is very little evidence that it can be achieved in real-world settings (Steenland et al., 2018). For example, in a recent stepped wedge trial in rural Nepal, households with LPG had a mean 20-h kitchen PM_2.5_ concentration of 442 μg/m^3^ and no differences in birth outcomes compared to houses with solid fuel stoves, likely due to continued use of solid fuels, air pollution from neighboring houses, and ambient pollution influenced in part by dust (Katz et al., 2020). Another recent trial in rural Ghana found that mothers randomized to receive LPG and cylinder refills had 32% lower mean 48-h PM_2.5_ personal exposure concentrations compared to women in the control arm (solid fuel) (Chillrud et al., 2021). However, the mean in the LPG arm was 52 ± 29 μg/m^3^, and two thirds of post-intervention maternal measurements in the LPG arm were above 35 μg/m^3^, possibly indicating the influence of ambient air quality and persistent solid fuel use (Chillrud et al., 2021).

The Household Air Pollution Intervention Network (HAPIN) Trial is a randomized controlled trial seeking to evaluate the health impacts of an LPG stove and fuel supply intervention on birthweight, stunting and severe pneumonia among children ≤1 year of age, and blood pressure among older women. The study is taking place in Guatemala, India, Peru, and Rwanda, and is currently ongoing (https://clinicaltrials.gov/ct2/show/NCT02944682 (Clasen et al., 2020)). As an efficacy trial, the aim is to maximize intervention uptake and use by providing the stove and fuel free of charge and by conducting comprehensive promotion and behavior change measures.

As part of formative research for the trial, numerous ambient, kitchen and personal PM_2.5_ concentration estimates have been conducted to assist in trial site selection and intervention design, as reported previously (Clasen et al., 2020; Sambandam et al., 2020). Other theory-grounded formative work has been conducted on participants’ perceptions of LPG, cooking behavioral differences across study sites, and the implementation components of the HAPIN trial, as well as development of strategies to monitor, promote and reinforce exclusive use of LPG for all cooking needs (Williams et al., 2020). Here we present results from a HAPIN pilot study in India, Rwanda, and Guatemala to estimate exposures to PM_2.5_ among pregnant women before and after a short-term LPG intervention, with the aim of characterizing baseline exposures and contrasts that might be expected as a result of the intervention in the main trial.

## 2 Methods

### 2.1 Study site and population

The HAPIN pilot intervention study was conducted in the countries where the main trial was proposed. Potential HAPIN sites were short-listed by country lead investigators in communities in India, Guatemala, Peru, and Rwanda. In Peru, the pilot intervention was conducted within the context of the Cardiopulmonary outcomes and Household Air Pollution trial (https://clinicaltrials.gov/ct2/show/NCT02994680) (Fandiño-Del-Rio et al., 2017), and the results have been published separately (Checkley et al., 2020).

Study sites were selected in rural areas, based primarily on the estimated proportion of households using biomass fuel; we additionally selected areas with relatively low levels of ambient air pollution, as determined during formative scoping activities preceding this pilot work. In India, we recruited from Villupuram and Nagapattinam Districts in Tamil Nadu; in Guatemala, from San Pedro Pinula and Jalapa municipalities from the Jalapa Department; and in Rwanda, from the Kabare, Murama and Kabarondo sectors in Kayonza District, Eastern Province.

### 2.2 Inclusion criteria

We enrolled 40 pregnant women in each study site. Pregnant women seeking routine antenatal care were screened at health centers and hospitals within study sites. The inclusion criteria were similar to those proposed for the main trial: 1) non-smoking pregnant women aged 18–34 years with gestational age 9–20 weeks confirmed through ultrasound (singleton pregnancy, without fetal anomalies or pregnancy complications identified at first ultrasound), 2) households using solid fuels as their main cooking fuel, and 3) no plans to move out of current household. Household selection among eligible houses was based on convenience and proximity to study offices in each study site. Details of household enrollment and follow-up are included in supplementary information (SI).

### 2.3 Study design and data collection

As part of HAPIN pilot phase, this study was designed as an LPG stove and fuel “before-and-after” single-arm, non-randomized intervention study (Sedgwick, 2014). For each household, we conducted one pre-intervention baseline visit and two follow-up visits (at approximately one- and two-months post-intervention), between July and November 2017. During each visit, we measured PM_2.5_ levels for 24 or 48 h (described below) and conducted household surveys using REDCap (Vanderbilt University, Nashville, TN, USA) to assess social demographic information, cooking practices and adherence of LPG intervention, compliance wearing the monitoring equipment, and to identify other potential sources of air pollution.

### 2.4 Intervention

Approximately 1 week after enrollment and baseline measurements, households received a new 2 or 3-burner gas stove, a regulator and hose, and a cylinder of gas that was refilled for free, upon demand, for two months. Cylinder fuel quantities were 15 kg in Rwanda, 14.2 kg in India, 11.3 kg in Guatemala. Upon receipt of the intervention, households completed a pledge to use it exclusively (Williams et al., 2020). Due to differences In perceptions of LPG use in Guatemala and Rwanda as compared to study sites in India, the intervention in Guatemala and Rwanda also included a behavior change component with messages tailored to address motivators and barriers to LPG use that was delivered in the local language, a demonstration of how to use the LPG stove safely, supervised practice with the stove, locally appropriate cooking tips, standardized videos showing how to use it, and printed educational and promotional materials that households could keep. Households were visited every 2–4 weeks (1) to answer any questions related to LPG stove use, (2) to reinforce behavior change messages, and (3) to provide cylinder refills as needed.

### 2.5 Air pollutant measurements

In each household, we conducted 24- or 48-h sampling of PM_2.5_ kitchen concentrations and personal exposures, once at baseline and twice at approximately around one- and two-months post-intervention, using active air samplers to estimate PM_2.5_ mass concentrations. Based on feasibility, 24-h sampling was conducted in Guatemala and Rwanda, while 48-h sampling was conducted in India. For kitchen area samples, instruments were placed in the same location each time for 24 or 48 h unless the kitchen location changed post-intervention: 1.5 m above ground, 1 m away from any doors and windows, and 1 m away from the combustion zone of the primary cookstove. For personal exposure samples, an apron or vest was designed at each site to carry the sampling devices, and participants were advised to wear this during the whole measurement period of 24 or 48 h. When bathing or sleeping, participants were instructed to place the apron/vest with the instruments less than 1 m away from them.

Due to logistical constraints, we used multiple instruments and size selective inlets to sample PM_2.5_ on either 15 mm (PT15-AN-PF02, MTL LLC., Minneapolis, MN USA) or 37 mm PTFE filters (Pall Life Sciences, Port Washington, NY, USA). We measured pump flow rates before and after sampling using a flow meter (Table 1). Filters were pre-weighed at temperature and humidity-controlled labs at Emory University (Atlanta GA, USA), University of Georgia (Athens GA, USA), Sri Ramachandra Institute of Higher Education and Research (Chennai, India), and Harvard University (Cambridge MA, USA). After sampling, filters were stored in a freezer and transported back under cold conditions to the same weighing lab where they are pre-weighed. In the total of 999 PM_2.5_ samples, we excluded PM_2.5_ samples whose average flow rate deviated from the desired flow rate by more than 10% (n = 7, 1%), whose sample duration deviated by more than 10% of the required duration (n = 38, 4%), and all filters with damage, such as holes or tears (n = 39, 4%), resulting in a total of 915 eligible gravimetric PM_2.5_ samples (kitchen area n = 413; personal exposure n = 502). Most of the eligible PM_2.5_ samples (n = 463, 51%) were collected on 15 mm filters using the Enhanced Children's MicroPEM (ECM, RTI International, Durham NC, USA), a robust, light gravimetric PM_2.5_ sampling instrument for the HAPIN trial with similar size to a cell phone. Additionally, 373 samples were collected on 37 mm filters using gravimetric pumps (SKC XR5000, Casella Air Tuff, and Casella TuffPro) and 79 samples on 37 mm filters using Access Sensor Technologies' (AST) Ultrasonic Personal Aerosol Sampler (UPAS) (Volckens et al., 2017). Detailed information regarding PM_2.5_ gravimetric instruments, filter weighing, blank filters, and sample quality assurance is in the supplementary information (SI).

**Table 1:** Details of gravimetric PM_2.5_ sampling instrument, number of samples, and filter weighing facilities in India, Guatemala, and Rwanda.

	India			Guatemala		Rwanda	
Instrument	SKC XR5000 pump, SKC PCXR8, Casella	AST UPAS^a^	RTI ECM	Casella Tuff pump	RTI ECM	Casella Tuff 3 Pro IS pump	RTI ECM
Size Selector	BGI Triplex cyclone	Built-in cyclone	Built-in impactor	BGI Triplex cyclone	Built-in impactor	H-PEM impactor (3243–3229)	Built-in impactor
Nominal flow rate (LPM)	1.5	1	0.3	1.5	0.3	1.8	0.3
Flow meter	Gilian Gilibrator-2 (Sensidyne) and TSI 41401			Gilian Gilibrator-2 (Sensidyne)		Gilian Gilibrator-2 (Sensidyne)	
Sampling Time (hour)	48	48	48	24	24	24	24
Filter diameter (mm)	37	37	15	37	15	37	15
Number of total eligible samples	8 (kitchen:8)	79 (kitchen:37; personal: 42)	133 (kitchen:57; personal: 76)	216 (kitchen:114; personal: 102)	230 (kitchen:121; personal: 109)	149 (kitchen:43; personal: 106	100 (kitchen:33; personal: 67)
Number of unique samples^b^	0	68 (kitchen:31; personal: 37)	133 (kitchen:57; personal: 76)	40 (kitchen:15; personal: 25)	193 (kitchen:104; personal: 89)	57 (kitchen:11; personal: 46)	100 (kitchen:33; personal: 67)
Sample duration (minutes)	2880			1440		1440	
Weighing facility	Sri Ramachandra Institute of Higher Education and Research (SRIHER)			Emory University, University of Georgia, Harvard University		Emory University, University of Georgia	

a UPAS: Ultrasonic Personal Air Sampler; ECM: Enhanced Children's MicroPEM; LPM: liters per minute.

b The difference between number unique samples and eligible samples is due to removal of co-located samples, with *a priori* using ECM instrument, gravimetric pumps (SKC and Casella) and then UPAS instruments.

Among all samples, 299 (72%) of 413 kitchen area samples were co-located samples (using two side-by-side sampling devices with the same operating time in the same location); 306 (61%) of 502 personal samples were co-located and collected with side-by-side PM_2.5_ samplers during the same household visit. ECMs were deemed as our *a priori* sampler and we only use ECM measurements from co-located samples, therefore, most of samples (N = 426) are from ECMs compared to other instruments (N = 165, Table 1). For any given household visit without ECM samples, we used samples collected by gravimetric pumps or UPAS instruments. Overall, we had 591 unique samples for following statistical analysis, comprised of kitchen area PM_2.5_ concentrations (N = 251) and pregnant women's personal exposure (N = 340). Among these samples, most were from ECM instruments (N = 426, 72%) and around a quarter were from gravimetric pumps (N = 97, 16%) and UPAS (N = 68, 12%).

The PM_2.5_ mass concentration for each sample was calculated using the average measured flow rates, the sample duration, and net change in mass of the filters (equation (1)).

(1) $$PM\;Conc.=\frac{\left(WT_{post}-WT_{pre}\right)-WTC_{BN}}{Flow\times Duration}\times 1000$$

PMConc. is the gravimetric PM_2.5_ concentration in μg/m^3^; $WT_{post}$ and $WT_{pre}$ are the post- and pre-weight of the filters in μg. $WTC_{BN}$ is the net filter mass change for blank filters (blanks were collected at each site in sampling pumps when they are turned-off). Flow is the sampler flow rate in L/min. In situations where the pre- or post-flow rate was not measured using a primary flow standard for ECMs, we used the average real-time flow rate logged by that ECM (N = 11, 2%). Duration is the sampling duration in minutes as recorded by field workers or logged by sampling devices.

We also collected 71 field blank filters spread out over the study period (India: 5 for 15 mm filters, 11 for 37 mm filters; Guatemala: 10 for 15 mm filters, 14 for 37 mm filters; Rwanda: 14 for 15 mm filters and 17 for 37 mm filters) to account for changes in filter mass due to handling and transportation. The median value of field blanks in Guatemala were 1.0 μg for 15 mm filters and −0.6 μg for 37 mm filters; in India, 0.3 μg for 15 mm filters and 0.75 μg for 37 mm filters; and in Rwanda, −0.25 μg for 15 mm filters and 10 μg for 37 mm filters.

### 2.6 Statistical analysis

Median, first, and third quartiles of kitchen gravimetric PM_2.5_ concentrations and personaPM_2.5_ exposures by each visit were summarized for 591 unique samples. We then used mixed effects linear models to estimate the reduction of kitchen area and personal PM_2.5_ after introduction of the LPG intervention. As kitchen PM_2.5_ measurements are log-normally distributed, we first log-transformed PM_2.5_ measurements, and checked normality of the distribution based on a quantile-quantile plot (Figure S1, SI). Then, we applied a linear mixed-effect model with household random intercepts to assess the reduction of kitchen PM_2.5_ concentrations and personal PM_2.5_ after LPG intervention. We included LPG intervention status (pre vs post), household characteristics, reported and observed sources of other air pollution during each household visit (self-reported smoke from cigarette smoking, trash burning, kerosene lighting, generators, smoking meat, and burning crops), and rainfall and temperature in the model (equation-2). Daily rainfall (in cm) and average temperature on the first day of each visit were determined by Climate Hazards Group InfraRed Precipitation with Station Data (CHIRPS) 2.0 (Funk et al., 2015) and Modern-Era Retrospective analysis for Research and Applications, Version 2 (MERRA -2, https://gmao.gsfc.nasa.gov/reanalysis/MERRA-2/) for the geographical centroid of each study site, respectively.

(2) $$\text{log}\left(PM_{ij}\right)=\beta_{0}+\beta_{1}LPG_{ij}+\beta_{2}X_{\mathrm{ij}}+\beta_{3}W_{i}+u_{i}+e_{ij}$$

$PM_{ij}$ is kitchen area PM_2.5_ or personal PM_2.5_ exposure for household *i* at time *j*; $\beta_{0}$ is the overall intercept, $LPG_{ij}$ indicates whether there is a LPG intervention for the measurement in household *i* at time *j*; $X_{\mathrm{ij}}$ includes time-dependent explanatory variables, including temperature, rainfall, and self-reported other sources of pollution during the measurement period; $W_{i}$ are time-independent explanatory variables, including study site, pregnant woman's age and years of education, her husband's years of education, and the number of people in the household. $u_{i}$ is the household random intercept of normal distribution with mean 0 and variance $\sigma_{b}^{2}$ , and $e_{ij}$ is the model residual following a normal distribution with mean 0 and variance $\sigma_{e}^{2}$. The random intercepts adjust for repeated measures from the same household where $\sigma_{b}^{2}$ refers to the between household variance and $\sigma_{e}^{2}$ refers to within household variance.

Subgroup stratified analysis were conducted for each site, and reductions of kitchen area and personal PM_2.5_ exposure were presented separately for each site. Additionally, we conducted sensitivity analysis for restriction to only households where kitchens locations were not changed after the intervention was delivered (SI). Data manipulation and processing were performed in R (version 3.6.0) and statistical analyses were performed in Stata (version 14.2). All relevant data and codebooks are publicly available (Liao et al., 2021).

### 2.7 Reduction of associated health effects

We modeled the potential health benefits for three of the primary outcomes assessed as part of the main HAPIN trial (Clasen et al., 2020): systolic blood pressure (SBP), birth weight, and as a proxy for severe pneumonia, acute lower respiratory infection (ALRI) by evaluating changes in outcomes or risk associated with reductions in the median personal PM_2.5_ exposure before and after LPG intervention based on state-of-the-science exposure-response curves (GBD, 2017 Risk Factor Collaborators, 2018; Steenland et al., 2018). Uncertainty interval (UI) bounds around these projected health effects were based on the interquartile range (IQR) of exposure estimates and 95% confidence interval of exposure-response curves.

### 2.8 Ethics

This pilot study received ethical approval from Emory IRB (00089,799), Universidad del Valle de Guatemala (146-08-2016/11–2016) and the Guatemalan Ministry of Health National Ethics Committee (11–2016), the Indian Council of Medical Research–Health Ministry Screening Committee (5/8/4–30/(Env)/Indo-US/2016-NCD-I), Sri Ramachandra Institute of Higher Education and Research (IEC-N1/16/JUL/54/49), London School of Hygiene and Tropical Medicine (11,664–2), and the Rwandan National Ethics Committee (No.148/RNEC/2017). Prior to participation, details of the study were provided to prospective participants and informed written consent was obtained.

## 3 Results

One hundred and twenty pregnant women from 120 households, were enrolled across all three countries and were followed from July through November 2017. Enrolled households were visited at baseline and for two follow-up visits after LPG fuel and stove intervention, with the exception of 1 household in Rwanda which exited the study prior to the first post-LPG exposure measurement visit.

### 3.1 Participant and household characteristics

Across the three countries, the mean age of pregnant women was 26.2 years (standard deviation 4.3) (Table 2). All participants were the primary cooks in their households. In Rwandan and Indian households, most women worked as farmers, while in Guatemala, housework was listed as the main occupation. At baseline, most of the households had fully enclosed kitchens; 28.3% (n = 34) of the households had unenclosed kitchens or cooked outdoors. Most households cooked with an open fire or built-in biomass stove without chimney (n = 104, 86.7%), or portable stoves with wood or charcoal (n = 10, 8.3%) (Table 2). All households used biomass (wood, grass/shrubs, charcoal or agriculture residue) as their primary fuel at baseline. After the LPG intervention, all open, outdoor kitchens were moved indoors (n = 70), typically within the house or to a separated kitchen. This change of kitchen location was mainly due to household preference and the feasibility of indoor LPG stove installation.

**Table 2:** Characteristics of enrolled pregnant women and households at baseline.

Variables	India	Guatemala	Rwanda	Overall
***Characteristics of women participants***				
	N = 40	N = 40	N = 40	N = 120
Age in years, mean (SD)	24.8 (3.3)	26.2 (4.2)	27.9 (4.3)	26.2 (4.3)
Years of schooling, pregnant woman, mean (SD)	7.5 (5.1)	3.65 (3.1)	5.6 (3.3)	5.6 (4.2)
Years of schooling, male partner, mean (SD)	7.4 (4.1)	4.15 (3.0)	6.6 (4.1)	6.1 (4.0)
Number of children under 12 years of age, mean (SD)	0.73 (0.7)	1.85 (1.4)	1.10(0.95)	1.21 (1.1)
Number of adults in household, mean (SD)	2.93 (1.25)	2.70 (1.7)	2.58 (1.2)	2.74 (1.4)
Employment status of pregnant woman				
Employed, n (%)	4 (10%)	38 (95%)	2 (5%)	44 (37%)
Unemployed or farmer, n (%)	36 (90%)	2 (5%)	38 (95%)	76 (63%)
Exposure to other sources of air pollution^a^, n (%)	11 (27.5%)	25 (62.5%)	15 (37.5%)	51 (42.5%)
***Household Characteristics***				
Kitchen type, n (%)				
Fully enclosed (roof with 4 walls)	32 (80%)	32 (82%)	22 (55%)	86 (71.7%)
Not enclosed/outdoor cooking	8 (20%)	8 (18%)	18 (20%)	34 (28.3%)
Fully enclosed kitchen measurements				
Kitchen size in m^2^, mean (SD)	9.9 (4.1)	12.5 (4.7)	5.2 (1.6)	10.0 (4.8)
Kitchen height in m, mean (SD)	2.8 (0.6)	2.53 (0.3)	2.58 (0.3)	2.64 (0.5)
# of doors, mean (SD)	1.40 (0.5)	1.15 (0.4)	1.14 (0.35)	1.24 (0.4)
# of windows or other openings, mean (SD)	1.82 (1.6)	7.06 (6.9)	1.23 (1.3)	3.63 (5.1)
Location of kitchen changed, n (%)	24 (60%)	10 (25%)	36 (90%)	70 (58%)
# of stoves, mean (SD)	1.31 (0.5)	1.47 (0.5)	1.33 (0.6)	1.37 (0.5)
Primary stove type, n (%)				
Open fire stove (three-stone)	40 (100%)	35 (87.5%)	11 (27.5%)	86 (71.7%)
Biomass stove with chimney	0	5 (12.5%)	0	5 (4.2%)
Rondereza (in-built wood-burning stove without chimney in Rwanda)	0	0	18 (45%)	18 (15%)
Portable stove (wood or charcoal)	0	0	10 (25%)	10 (8.3%)
Other	0	0	1 (7.5%)	1 (2.4%)
Primary stove fuel type, n (%)				
Grass/Shrubs	0	0	9 (22.5%)	9 (7.5%)
Agricultural waste	0	0	1 (2.5%)	1 (0.8%)
Wood	40 (100%)	40 (100%)	25 (62.5%)	105 (87.5%)
Charcoal	0	0	5 (12.5%)	5 (4.2%)
# of burners of primary stove, n (%)				
1	4 (9.7%)	28 (70%)	29 (72.5%)	61 (50.1%)
2	36 (90.3%)	4 (10%)	9 (22.5%)	49 (40.8%)
≥3	0	8 (20%)	2 (5%)	10 (4.2%)
***Meteorological Characteristics***				
Average Rainfall (cm)	6.1	5.7	1.5	4.5
Average temperature (°C)	29.3	22.9	22.4	25.0

a Self-reported exposure to second-hand smoke, mosquito coil burning, trash burning, kerosene burning, incense burning, electricity generator, smoking meat, or crop burning over the past 24 h.

### 3.2 LPG use

Households had high compliance with the LPG intervention, according to field worker observation (at end of measurement period) and self-reported fuel use during the 24/48-h measurement period. Only 3 out of 226 household visits indicated biomass use after receipt of the intervention, and all were from the second follow-up approximately two months after receipt (2 households from Guatemala and 1 from Rwanda).

### 3.3 PM_2.5_ concentrations

**Fig. 1:**
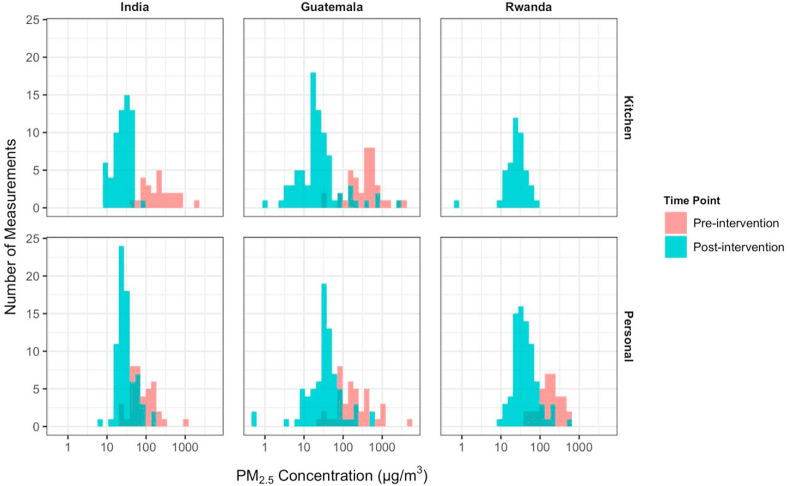
Distributions of kitchen PM_2.5_ concentrations and personal PM_2.5_ exposure before (biomass) and after LPG intervention for 591 Samples.

We show the distribution of log-transformed 24/48-h kitchen area and personal PM_2.5_ concentrations by study site and LPG intervention status in Fig. 1. Only Guatemala and India conducted baseline kitchen PM_2.5_ measurements (in a total of 65 households). Ninety-four and 92 kitchen area measurements were collected from all three sites, from follow-up 1 and follow-up 2 visits, respectively.

Baseline personal PM_2.5_ exposure measurements were conducted in three sites. We found that the LPG fuel and stove intervention resulted in a substantial reduction of both kitchen level and personal PM_2.5_ concentrations, in 591 samples removing duplicates (Table 3). The median (Q1-Q3) kitchen level PM_2.5_ concentrations were 296 (158–507) μg/m^3^ at baseline, 24 (18–37) μg/m^3^ at follow-up 1 and 23 (14–37) μg/m^3^ at follow-up 2 after LPG intervention. The median (Q1 - Q3) personal PM_2.5_ exposure were 134 (71–224) μg/m^3^ at baseline, 35 (23–51) μg/m^3^ at follow-up 1 and 32 (23–47) μg/m^3^ at follow-up 2. The complete samples of gravimetric PM_2.5_ measurements (N = 915) with duplicates showed similar reductions of kitchen area PM_2.5_ and personal PM_2.5_ exposure (Table S1 in SI).

**Table 3:** Summary statistics (median, first quartile, third quartile) of kitchen and personal PM_2.5_ levels (μg/m^3^) for 591 unique samples by each visit and site.

		Baseline			Follow-up 1			Follow-up 2		
		N	Median	Q1 – Q3	N	Median	Q1 – Q3	N	Median	Q1 – Q3
India*	Kitchen (48 h)	26	191	96–368	30	20	14–31	32	32	23–38
	Personal (48 h)	40	71	50–134	38	26	20–37	35	28	24–38
Guatemala	Kitchen (24hr)	39	433	193–511	40	27	19–37	40	15	7–24
	Personal (24 h)	39	151	86–345	37	38	30–45	38	30	13–60
Rwanda	Kitchen (24hr)				24	26	20–36	20	25	19–37
	Personal (24 h)	35	175	112–276	39	46	26–67	39	36	26–49
All	Kitchen	65	296	158–507	94	24	18–37	92	23	14–37
	Personal	114	134	71–224	114	35	23–51	112	32	23–47

N: number of measurements, Q1: first quartile, Q3: third quartile; grey shaded area indicates no measurements were conducted.

*Estimates presented here are derived from the same database as those reported by Sambandam et al. (2020). The number of measurement (N) diverges slightly due to different exclusion criteria, as we excluded samples that deviated more than 20% of target length of time or samples with filter damage, differing slightly to exclusion criteria used by Sambandam et al. (2020).

### 3.4 Reduction of personal PM_2.5_ exposure from LPG intervention

In Table 4, we show, using mixed effect linear models, that the LPG intervention is associated with a 92% (95% CI: 90%–94%) reduction of kitchen PM_2.5_ concentrations and a 74% (95% CI: 70%–79%) reduction of personal PM_2.5_ exposures. Reductions of kitchen and personal PM_2.5_ after LPG intervention are similar across sites. We observed a higher reduction of kitchen PM_2.5_ concentrations compared to personal PM_2.5_ exposures, with the highest reductions of PM_2.5_ in Guatemalan kitchens. In the sensitivity analysis including only households where kitchen locations have not changed (N = 50), the reduction due to the LPG intervention is similar (SI).

**Table 4:** Reduction (95% confidence interval) of kitchen PM_2.5_ concentration and personal PM_2.5_ exposure with LPG intervention.^a^.

Model	All three sites	Stratified by site		
		India	Guatemala	Rwanda
Kitchen PM_2.5_	92% (90%–94%)	90% (86%–93%)	97% (90%–99%)	n/a
Personal PM_2.5_	74% (70%–79%)	58% (45%–67%)	83% (76%–88%)	70% (60%–77%)

a Based on linear mixed effects models, adjusted by study site, pregnant woman's age and years of education, husband's year of education, the number of people in the household, temperature, rainfall, and self-reported other sources of air pollution during the measurement period. Kitchen PM_2.5_ concentrations and personal PM_2.5_ exposures are log-transformed; grey shaded area indicates no analysis was conducted.

**Fig. 2:**
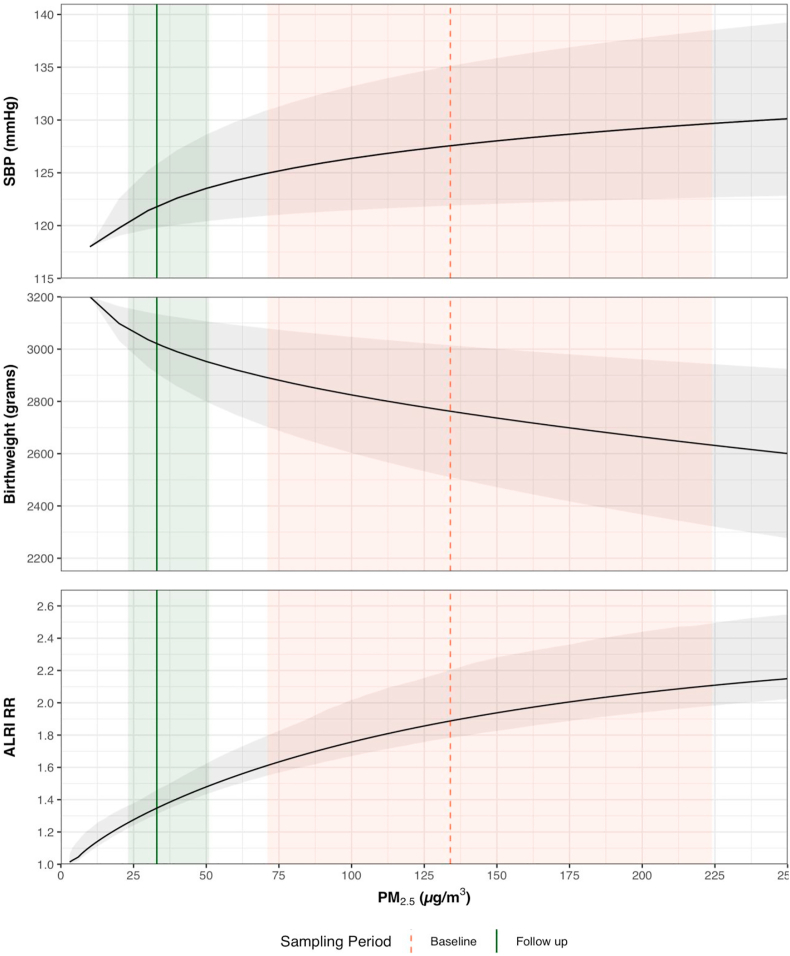
Potential effects of changes in PM_2.5_ personal exposure on systolic blood pressure (SBP) and birth weight (BW) (both adapted from Steenland et al., 2018) and relative risk (RR) of acute lower respiratory infection (ALRI) (adapted (GBD, 2017 Risk Factor Collaborators, 2018). Across all panels, the modeled exposure response curve is shown as a solid black line with uncertainty intervals in light grey. The pink dashed line is the median personal exposure during the baseline period; the green solid line is the median personal exposure during the follow-up period after LPG intervention. The correspondingly colored shaded areas are the IQR of measurements during that period. (For interpretation of the references to color in this figure legend, the reader is referred to the Web version of this article.)

As shown in Fig. 2, observed personal exposures after LPG intervention suggest transitioning to LPG in these settings could result in health benefits. Given the exposure reductions we observed, we estimate a reduction of 5.7 mmHg (uncertainty interval (UI): 5.1–6.1) in SBP after LPG, a 250 g (UI 205–315) increase in birth weight; and a reduction in ALRI relative risk from 1.8 (1.6–2.1) to 1.4 (1.3–1.5).

## 4 Discussion

### 4.1 Context of HAP exposures

The introduction of LPG stoves and fuel in primarily biomass-using households, accompanied by behavior change strategies, was associated with substantial reductions in both kitchen concentrations of and personal exposures to PM_2.5_ in three countries. Effects were consistent for two follow-up measurements one month apart, and overall the intervention yielded median kitchen and personal concentrations at, or lower than the annual WHO interim target-1 (IT-1) of 35 μg/m^3^ for the first and second follow-ups. The effect was most pronounced in Guatemala for both kitchen concentrations and personal exposures, potentially due to low ambient pollution and the limited impacts of other sources of exposure, as well as high baseline PM_2.5_ levels. Only 3 households (2.5%) reported biomass stove use after receiving the LPG intervention. Although relying on reported stove and fuel use is not as accurate as direct measurement using stove use monitors, the lack of observed traditional stove use when field workers arrived and anecdotal reports from field staff suggests that LPG was likely used nearly exclusively.

Variability in kitchen concentrations and exposures was evident between field sites, with higher PM_2.5_ levels in Rwanda and Guatemala, compared to those measured in India. This type of variation has been reported in systematic HAP review papers (Pope et al., 2017; Quansah et al., 2017), and is similar to the pattern observed in the recent Prospective Urban and Rural Epidemiological (PURE) study, which also reported higher PM_2.5_ exposures for women in biomass-using homes in Africa (geomean = 153 μg/m^3^) compared to India (geomean = 89 μg/m^3^) (Shupler et al., 2020). There are several reasons for regional variation in HAP exposures due to differences in environmental and behavioral factors, such as location of cooking (indoors/outdoors), quantity of cooking and/or fuel use, proximity to neighbors, and others. Here, we speculate that all these factors likely contributed to the differences of HAP we observed.

The relative reductions in PM_2.5_ were greatest for kitchens, which is unsurprising given stoves are the dominant source in that environment. Our observed kitchen reductions are similar to a study in Cameroon (LACE-1), which found significant differences between exclusively biomass using households and those using LPG, with median kitchen concentrations of 447.9 μg/m^3^ and 21.1 μg/m^3^ respectively (Bruce et al., 2018). Personal exposures among women from the same study were 46.9 μg/m^3^ and 14.0 μg/m^3^ respectively, although the relatively low exposures observed may have been due to season and behavior, including minimal time spent in kitchen (Bruce et al., 2018). In general, personal exposure levels were slightly higher than kitchen levels during the post-intervention period, and kitchens had more dramatic reductions, perhaps indicating other sources of exposure experienced outside of kitchens. Similarly, a recent study in India found that switching to LPG resulted in an 85% reduction in kitchen PM_2.5_, but mean levels were only reduced to 76 μg/m^3^ (approximately twice the annual interim target), potentially due to unmeasured traditional stove use and ambient air pollution (Pillarisetti et al., 2019b). A larger proportional reduction in kitchen rather than personal exposure was also observed in a wood-burning chimney stove intervention in Guatemala (Smith et al., 2010). We observed a similar trend (higher personal vs kitchen after intervention) most prominently in Rwanda at the first round of follow-up, although the second round saw further reductions in personal exposure which approached kitchen levels.

In settings of low ambient air pollution and near-exclusive stove uptake, both kitchen and personal exposure levels of PM_2.5_ can approach or meet health-based WHO targets, as observed in a recent LPG trial in Peru where 23% of kitchens and 78% of personal measurements were less than 35 μg/m^3^ in the intervention arm (Checkley et al., 2020). By contrast, clean fuel use within a context of non-exclusive use, neighboring traditional stove use, and higher ambient levels can limit exposure reduction potential, as observed in the GRAPHS trial where two thirds of post intervention maternal measurements were above 35 μg/m^3^ in the intervention arm (Chillrud et al., 2021). Similarly, a trial in Nigeria found limited personal exposure contrasts between mothers assigned to ethanol compared to kerosene and wood-burning stoves, with mean 72-h PM_2.5_ means of 118 (SD 166) μg/m^3^ vs. 102 (SD 102) μg/m^3^ respectively in the dry season, and 61 (SD 74) vs 66 (SD 82) μg/m^3^ respectively in the rainy season (Alexander et al., 2018). More dramatically in rural Nepal, households assigned to LPG had a mean 20-h kitchen concentration above 400 μg/m^3^ (Katz et al., 2020).

Our PM_2.5_ findings further suggest the capacity to reach the WHO targets with a clean fuel intervention, and are generally similar to, or lower than other estimates of PM_2.5_ among LPG-using households in these regions. Recent modeled global and regional estimates for kitchen and personal PM_2.5_ across different fuel types in rural areas indicate that our results are similar or in some cases lower (Shupler et al., 2018). Globally, it is estimated that transitioning from traditional cooking with wood to gas would reduce mean (95% CI) of kitchen concentrations from 395 (148, 1039) μg/m^3^ to 105 (39, 273) μg/m^3^ (Shupler et al., 2018), and the PURE study, conducted across 8 countries, found that mean kitchen concentrations among gas-using households was 45 μg/m^3^ (95% CI 43–48), 53 μg/m^3^ among electricity-using households, and above 90 μg/m^3^ among charcoal, wood, and agricultural/crop waste-burning households, and over 200 μg/m^3^ among animal dung and shrub/grass-burning households (Shupler et al., 2020). Encouragingly, a recent stepped wedge intervention trial in Honduras comparing traditional wood-burning cooking to a wood stove with an improved combustion chamber and chimney (*Justa*) had substantially lower kitchen PM_2.5_ and personal exposure concentrations with means below 80 and 40 respectively, and with over 40% of personal measures falling below 37.5 μg/m^3^ (Benka-Coker et al., 2021).

### 4.2 Implications for HAPIN trial

Exposure reductions fell on steep parts of currently estimated exposure-response curves for several important health outcomes (Bruce et al., 2015; Burnett et al., 2014; Steenland et al., 2018). Therefore, based on the exposure-response curves, we should be able to observe health benefits associated with LPG intervention if the reduction of personal PM_2.5_ is consistently maintained across a longer observational period. Furthermore, based on these exposure-response curves, even reductions of personal PM_2.5_ exposures above 35 μg/m^3^, the annual IT-1 guideline, would also be expected to deliver some health benefits, particularly for blood pressure, birthweight, and ALRI (Steenland et al., 2018). Another published PM_2.5_-mortality hazard ratio function based on outdoor air pollution in multiple countries suggests these reductions may lower risk of mortality as well (Burnett et al., 2018). However, reducing kitchen and personal exposures below WHO interim guidelines through a stove intervention does not guarantee health benefits will be observed, as reported in a recent trial in rural Peru which found no impacts among 180 women aged 25–64 on systolic or diastolic blood pressure, peak expiratory flow, or respiratory symptoms (Checkley et al., 2020). Furthermore, there was no exposure-response relationship found between personal exposure to HAP and primary blood pressure outcomes (Checkley et al., 2020). Reasons for this may include small sample size, a relatively healthy population that under studied, and limited length of time during which exposure reductions were sustained and health effects were studied. This suggests that longer follow-up may be necessary, especially for chronic disease outcomes (Checkley et al., 2020; Kaali et al., 2020). Furthermore, persistence of other pollutants present when cooking with gas, such as NO_2_, may minimize potential health benefits (Kephart et al., 2021). Given the variability and uncertainty in exposure-response relationships, exposures may need to be much lower than WHO interim targets and sustained over longer periods of the time in order to sufficiently achieve meaningful health effects (Kaali et al., 2020).

Thus, while this pilot study's PM_2.5_ exposure contrasts fell on promising sections of published exposure-response curves (e.g. Fig. 2), the potential for clean cooking interventions to meaningfully improve health remains to be demonstrated and these curves may be updated as more observational studies and trials are conducted. Trials such as HAPIN are thus needed to address important gaps in the evidence for health benefits of cleaner cooking, as well as help better characterize exposure-response curves for several health outcomes that contribute substantially to morbidity and mortality globally (Clasen et al., 2020).

### 4.3 Limitations

This study has a number of limitations. Perhaps most important, as pilot work, the sample sizes were relatively small, and although we found significant differences, the kitchen concentration and exposure estimates are not highly robust. Further, the convenience sample and before-and-after single-arm, non-randomized intervention design without a parallel control group leaves open the possibility that effects were due in part to selection bias and unmeasured time-varying confounders. Particularly, seasonal differences in environmental conditions and household practices could have contributed to observed differences, although we controlled for temperature and precipitation in an effort to account for these changes, and there was little variation between baseline and follow-up in each study site. The short length of follow-up time over which air quality monitoring was conducted is another limitation, even though this can minimize impact of season and other temporal trends of ambient and household PM_2.5_ levels. Use of the intervention stove may decrease over time (Pillarisetti et al., 2014), and traditional stove use may increase, especially for certain cooking tasks where traditional stoves may be preferred (Ruiz-Mercado and Masera, 2015). Certain cooking tasks and stove preferences may vary seasonally, and other seasonal activities such as heating can also impact exposure. A study in rural China found that personal exposure was only 37% lower among those who primarily used LPG or electricity compared to biomass users, although this was only observed in summer when heating was not as common (Ni et al., 2016). We speculate that unmeasured confounders across sites and visit periods contributed to relative differences in kitchen concentrations of and personal exposures to PM_2.5_ post-intervention; however, we are not able to test these hypotheses due to limited contextual data we were able to feasibly collect, and small sample sizes.

In this study, we did not systematically measure traditional stove usage with stove use monitors, which could strengthen the evidence that LPG uptake was near-exclusive and sustained, although the lack of observed traditional stove use by field workers supports high uptake of the intervention, which was also supported by low self-reported traditional stove post-intervention. It is possible that self-reported traditional stove use is under-reported due to response bias, which could partially explain the small number of higher PM_2.5_ concentrations, especially for kitchen measurements. Those higher kitchen concentrations and exposure estimates during follow-up measurements may also be the result of other factors such as exposure to neighbor's smoke, trash-burning, or others. It is also possible that the measurements are not representative of normal exposures due to the Hawthorne effect, with more exclusive use of LPG during the 24–48 h of monitoring compared when HAP monitoring wasn't occurring (Lozier et al., 2016). Additionally, real-time compliance of wearing the exposure monitoring equipment was also not measured. The HAPIN main trial will provide an opportunity to look at LPG and traditional stove use over an 18-month period using sensors, along with exposure equipment-wearing compliance using accelerometers, and will thus be able to more objectively and accurately characterize stove use and exposure reductions observed over time (Johnson et al., 2020).

Another limitation of our study is the application of different PM_2.5_ measurement instrumentation at the field sites. While the different instrumentation was a necessity given the resources and timing of the pilot study, protocols were the same across research sites in terms of filter handling, flow checks, microbalance specifications and weighing procedures, and others key to the collection of gravimetric PM_2.5_ samples. Moreover, instrument comparison studies, including one by the HAPIN study team, demonstrated similar performance between ECMs, UPASes, and pump and filter systems (Arku et al., 2018; Burrowes et al., 2020; Du et al., 2019; Pillarisetti et al., 2019a; Volckens et al., 2017).

We only measured primary cooking area PM_2.5_ concentrations and only personal exposure experienced by pregnant woman. While these are expected to be representative of what a clean stove intervention could achieve in terms of kitchen and personal PM_2.5_ reductions among primary cooks, future work will incorporate monitoring of multiple household environments, children and other household members in order to provide better PM_2.5_ exposure estimates (Johnson et al., 2020; Liao et al., 2019).

## 5 Conclusion

The pilot results of this LPG intervention as implemented in three different countries show promise for reducing exposure to levels expected to deliver health benefits (Burnett et al., 2014; Steenland et al., 2018). Ongoing and soon-to-be published clean cookstove studies conducted in Ghana will provide further evidence of the potential of similar interventions to improve health and sustain uptake (Carrión et al., 2018; Jack et al., 2015). Further research is needed to examine if effects and near-exclusive use are sustained over time (Rosenthal, 2015), if health benefits are in fact achieved (Ezzati and Baumgartner, 2016; Thakur et al., 2018), and how barriers to clean cooking can be reduced at household, community and national levels (Puzzolo et al., 2016), particularly among the poorest (Brooks et al., 2016).

## Author statement

Jiawen Liao: Conceptualization, Methodology, Software, Formal analysis, Data Curation, Writing, Writing - Review & Editing, Miles Kirby: Conceptualization, Methodology, Software, Formal analysis, Investigation, Data, Writing Curation, Writing - Review & Editing, Supervision, Ajay Pillarisetti: Conceptualization, Methodology, Data Curation, Writing, Writing - Review & Editing, Ricardo Piedrahita: Methodology, Data Curation, Writing, Kalpana Balakrishnan: Conceptualization, Methodology, Writing - Review & Editing, Supervision, Project administration, Funding acquisition, Sankar Sambandam: Investigation, Data Curation, Supervision, Krishnendu Mukhopadhyay: Investigation, Data Curation, Supervision, Wenlu Ye: Formal analysis, Data Curation, Ghislaine Rosa: Conceptualization: Investigation, Supervision, Project administration, Funding acquisition, Fiona Majorin: Investigation, Supervision, Ephrem Dusabimana: Investigation, Supervision, Florien Ndagijimana: Investigation, Supervision, John P. McCracken: Conceptualization, Methodology, Writing - Review & Editing, Supervision, Project administration, Erick Mollinedo: Investigation, Data Curation, Oscar de Leon: Formal analysis, Investigation, Data Curation, Anaité Díaz-Artiga: Investigation, Supervision, Lisa M. Thompson: Writing - Review & Editing, Supervision, Project administration, Katherine Kearns: Data Curation, Luke Naeher: Methodology, Writing - Review & Editing, Joshua Rosenthal: Conceptualization, Writing - Review & Editing, Maggie L. Clark: Writing, Writing - Review & Editing, Kyle Steenland: Methodology, Writing, Writing - Review & Editing, Lance A. Waller: Formal analysis, Writing - Review & Editing, William Checkley: Conceptualization, Writing - Review & Editing, Project administration, Funding acquisition, Jennifer L. Peel: Conceptualization, Writing - Review & Editing, Project administration, Funding acquisition, Thomas Clasen: Conceptualization, Writing - Review & Editing, Project administration, Funding acquisition, Michael Johnson: Conceptualization, Methodology, Writing, Writing - Review & Editing, Project administration.

## Declaration of competing interest

The authors declare that they have no known competing financial interests or personal relationships that could have appeared to influence the work reported in this paper.
