# Supplementary material for: LPG stove and fuel intervention among pregnant women reduce fine particle air pollution exposures in three countries: Pilot results from the HAPIN trial
Source: Environ Pollut. 2021 Dec 15;291:118198. doi: 10.1016/j.envpol.2021.118198 (PMC8593210; doi:10.1016/j.envpol.2021.118198)

Supplementary Information

**LPG stove and fuel intervention among pregnant women reduce fine particle air pollution exposures in three countries: Pilot results from the HAPIN Trial**

**Jiawen Liao^1^, Miles Kirby^1, 2^** **(co-first)**, Ajay Pillarisetti^1, 3^, Ricardo Piedrahita^4^, Kalpana Balakrishnan^5^, Sankar Sambandam^5^, Krishnendu Mukhopadhyay^5^, Wenlu Ye^1^, Ghislaine Rosa^6^, Fiona Majorin^6^, Ephrem Dusabimana^7^, Florien Ndagijimana^7^, John P. McCracken^8^, Erick Mollinedo^8^, Oscar de Leon^8^, Anaité Díaz-Artiga^8^, Lisa M. Thompson^1, 9^, Katherine Kearns^10^, Luke Naeher^10^, Joshua Rosenthal^11^, Maggie L. Clark^12^, Kyle Steenland^1^, Lance A. Waller^1^, William Checkley^13, 14^, Jennifer L. Peel^12^, Thomas Clasen^1^, Michael Johnson*^4^, HAPIN Investigators

^1^ Emory University Rollins School of Public Health, Department of Environmental Health, Atlanta, GA, USA

^2^ Harvard T.H. Chan School of Public Health, Boston, MA, USA

^3^ School of Public Health, University of California, Berkeley, CA, USA

^4^ Berkeley Air Monitoring Group, Berkeley, CA, USA

^5^ SRU-ICMR Center for Advanced Research on Air Quality, Climate and Health, Sri Ramachandra Institute of Higher Education and Research, Chennai, India

^6^ London School of Hygiene and Tropical Medicine, London, UK

^7^ Eagle Research Center, Kigali, Rwanda

^8^ Center of Health Studies, de Estudios en Salud, Universidad del Valle De Guatemala, Guatemala City, Guatemala

^9^ Nell Hodgson Woodruff School of Nursing, Emory University, Atlanta, GA, USA

^10^ College of Public Health, University of Georgia, Athens, GA, USA

^11^ Fogarty International Center, National Institutes of Health, Bethesda, MD, USA

^12^ Department of Environmental and Radiological Health Sciences, Colorado State University, Fort Collins, CO, USA

^13^ Division of Pulmonary and Critical Care, School of Medicine, Johns Hopkins University, Baltimore, MD, USA

^14^ Center for Non-Communicable Disease Research and Training, School of Medicine, Johns Hopkins University, Baltimore, MD, USA

*Corresponding Author: Michael A. Johnson, PhD, mjohnson@berkeleyair.com

**Gravimetric PM_2.5_ sampling, Filter Weighing**

The PM_2.5_ sampling instruments varied by study site, but applied similar mechanisms to collect particulate matter with aerodynamic diameter of less than 2.5 micrometer on filters, with sampling times of either 24 or 48 hours. At the India site, we used Casella Tuff Pro (Casella Measurement, Bedford, UK), Airchek XR5000 and Universal PCXR8 pumps (SKC, Eighty Four, PA, USA) connected to a Triplex personal sampling cyclone (SCC 1.062, BGI, Cambridge, MA, USA); Ultrasonic Personal Air Sampler (UPAS, Colorado State University, Fort Collins CO, USA) with built-in cyclone (Volckens et al. 2017); and Enhanced Children’s MicroPEM (ECM, RTI International, Durham, NC, USA) with built-in impactor. Pallflex membrane 37mm filters (Pall Life Sciences, Port Washington, NY, USA) and PTFE membrane 15mm filters (PT15-AN-PF02, MTL LLC., Minneapolis, MN USA) were used. At the Guatemala site, we used Casella Tuff pumps (Casella, Bedford, United Kingdom) with BGI Triplex cyclones (SCC 1.062, BGI, Cambridge, MA, USA) and Enhanced Children’s MicroPEM (ECM, RTI, Research Triangle Park, NC, USA) with built in impactor. PTFE membrane 37mm filters (Pall Life Sciences, Port Washington, NY, USA) and PTFE membrane 15mm filters (PT15-AN-PF02, MTL LLC., Minneapolis, MN USA) were used. In Rwanda, we used Casella TuffPro pumps (Casella Measurement, Bedford, UK) with Harvard Personal Exposure Monitor (H-PEM) impactors (BGI, Cambridge, MA, USA). PTFE membrane 37mm filters (Pall Life Sciences, Port Washington, NY, USA) and PTFE membrane 15mm filters (PT15-AN-PF02, MTL LLC., Minneapolis, MN USA) were used. Pump flow rates for each device were calibrated in an office/lab setting prior to deployment using Gilian Gilibrator-2 (St. Petersburg, Florida, USA) at each site, and additionally the Mass Flow Meter (41401, TSI Inc., Minnesota, USA) at the India site. After the 24 or 48 hour measurement period, filters were stored in petri dishes and placed in office refrigerators and shipped under cold conditions with ice packs (at temperatures below 25°C). The blank filter samples were collected at each study site (India, Guatemala, and Rwanda) based on the sample protocol. Specifically, blank filters are placed in the sampling instrument the same as other filters, are taken to the field but without turning on the pump.

The filters were pre- and post-weighed in multiple labs (Emory University, Atlanta, Georgia; University of Georgia, Athens, Georgia; Harvard University, Cambridge, Massachusetts; SRU-ICMR Center for Advanced Research on Air Quality, Climate and Health, Sri Ramachandra Institute of Higher Education and Research, Chennai, India). All filters were conditioned for 24 hours before weighing and were post-weighed in the same lab in which they were pre-weighed. All filter weighing labs followed a similar protocol of temperature and humidity control with temperatures between 20 - 24°C and relative humidity between 30% - 50%. For each filter at pre- and post-weighing, two weight measurements were made, with a third weight taken if the first two weights differed by more than 5 μg.

**Household Screening and Enrollment**

120 households were enrolled across all three countries. Enrollment began in July and August 2017: India (18 July 2017), Guatemala (31 July 2017), Rwanda (2-21 August 2017). In India, 103 women were screened and 40 were enrolled. Reasons for not becoming enrolled included mixed fuel/LPG usage (n=24), unwilling or incomplete ultrasound screening (n=23), ineligible according to ultrasound reading (n=12) or refusal to participate (n=4). In Rwanda, 79 women were screened and 40 were enrolled; reasons for not being enrolled include ineligible according to ultrasound readings (n=30), ineligible according to screening criteria (n=5), and target sample size already reached (n=4). In Guatemala, 104 women were screened and 40 were enrolled; reasons for not being enrolled included refusal to participate (n=3), LPG or chimney stove usage (n=2), BMI > 40kg/m2 (n=1), and ineligible according to ultrasound readings (n=58). All enrolled households were visited at baseline and for two follow-up visits, with the exception of 1 house in Rwanda which exited the study prior to the first post-LPG exposure visit due to premature delivery. Rwanda baseline took place from 10-25 August 2017, follow-up 1 from 20 September-7 October 2017, and follow-up 2 from 23 October-11 November 2017. India baseline took place 25 July-22 August 2017, follow-up 1 from 17 August– 03 September 2017, and follow-up 2 from 3 September– 26 October 2017. Guatemala baseline took place 01 August – 26 August 2017, follow-up 1 from 28 August – 07 September 2017, and follow-up two from 20 September – 26 October 2017.

**Sensitivity Analysis with Household without Moving Kitchen Locations**

We conducted sensitivity analysis only for data from 50 households without moving kitchen locations, and assess the reduction of kitchen area PM_2.5_ levels (N = 124) and personal PM_2.5_ exposures (N =141), with the same model specification as main analysis. The sensitivity analysis shows LPG is associated with 93% (95% CI: 91% - 95%) reduction in kitchen area PM_2.5_ concentrations, and with 82% (95% CI: 76% - 86%) reduction in personal PM_2.5_ exposure. This translates to 264.7 µg/m^3^ (95% CI: 259.2 – 270 µg/m^3^) reduction in kitchen area PM_2.5_ concentrations and 126 µg/m^3^ (95% CI: 117 - 132 µg/m^3^) reduction in personal PM_2.5_ exposures.

References

Volckens J, Quinn C, Leith D, Mehaffy J, Henry CS, Miller-Lionberg D. 2017. Development and evaluation of an ultrasonic personal aerosol sampler. Indoor Air 27:409–416; doi:10.1111/ina.12318.

Table S1. Kitchen Area and Women’s Personal PM_2.5_ Assessment before and after HAPIN Pilot LPG Intervention, including all eligible samples (N = 915)

|  |  |  | **Baseline** | | | | **Follow-up 1** | | | | **Follow-up 2** | | | |
| --- | --- | --- | --- | --- | --- | --- | --- | --- | --- | --- | --- | --- | --- | --- |
|  |  | **Device** | **N_m_** | **N_hh_** | **Median** | **Q1 – Q3** | **N_m_** | **N_hh_** | **Median** | **Q1 – Q3** | **N_m_** | **N_hh_** | **Median** | **Q1 – Q3** |
| **India** | **Kitchen (48 hr)** | ECM | 17 | 17 | 186 | 93 – 390 | 19 | 19 | 21 | 15 – 37 | 21 | 21 | 32 | 22 – 39 |
|  |  | SKC | 3 | 3 | 39 | 27 – 44 | 2 | 2 | 17 | 16 – 18 | 3 | 3 | 32 | 26 – 77 |
|  |  | UPAS | 11 | 11 | 159 | 99 – 253 | 13 | 13 | 18 | 14 – 22 | 13 | 13 | 28 | 25 – 37 |
|  |  | **ALL** | **31** | **26** | **160** | **92 – 298** | **34** | **31** | **18** | **15 – 30** | **37** | **32** | **32** | **23 – 28** |
|  | **Personal (48 hr)** | ECM | 27 | 27 | 97 | 56 – 162 | 25 | 25 | 31 | 21 – 48 | 24 | 24 | 27 | 23 – 38 |
|  |  | SKC |  |  |  |  |  |  |  |  |  |  |  |  |
|  |  | UPAS | 15 | 15 | 57 | 47 – 77 | 15 | 15 | 22 | 19 – 32 | 12 | 12 | 28 | 24 - 34 |
|  |  | **ALL** | **42** | **40** | **71** | **50 – 130** | **40** | **38** | **26** | **21 – 37** | **36** | **35** | **28** | **23 - 38** |
| **Guatemala** | **Kitchen (24 hr)** | ECM | 37 | 30 | 383 | 176 – 508 | 39 | 39 | 27 | 19 – 37 | 45 | 35 | 15 | 7 – 25 |
|  |  | Casella | 43 | 35 | 240 | 172 – 449 | 30 | 30 | 12 | 5 – 21 | 41 | 33 | 6 | 3 – 11 |
|  |  | UPAS |  |  |  |  |  |  |  |  |  |  |  |  |
|  |  | **ALL** | **80** | **39** | **305** | **171 - 496** | **69** | **40** | **21** | **13 – 31** | **86** | **40** | **9** | **5 – 22** |
|  | **Personal (24 hr)** | ECM | 39 | 29 | 151 | 88 – 255 | 30 | 30 | 37 | 30 – 46 | 40 | 30 | 33 | 20 – 74 |
|  |  | Casella | 36 | 36 | 100 | 60 – 339 | 30 | 30 | 20 | 12 – 39 | 36 | 36 | 8 | 4 – 28 |
|  |  | UPAS |  |  |  |  |  |  |  |  |  |  |  |  |
|  |  | **ALL** | **75** | **39** | **141** | **77 – 296** | **60** | **37** | **33** | **18** – **44** | **76** | **38** | **23** | **8** – **62** |
| **Rwanda** | **Kitchen**  **(24 hr)** | ECM |  |  |  |  | 15 | 15 | 25 | 20 – 32 | 18 | 18 | 26 | 20 – 37 |
|  |  | Casella |  |  |  |  | 23 | 23 | 27 | 19 – 46 | 20 | 20 | 27 | 15 – 39 |
|  |  | UPAS |  |  |  |  |  |  |  |  |  |  |  |  |
|  |  | **ALL** |  |  |  |  | **38** | **24** | **27** | **20 - 38** | **38** | **20** | **26** | **18 – 37** |
|  | **Personal**  **(24 hr)** | ECM | 35 | 35 | 175 | 112 – 277 | 29 | 29 | 43 | 25 – 58 | 38 | 38 | 35 | 26 – 45 |
|  |  | Casella | 11 | 11 | 146 | 100 – 170 | 35 | 35 | 81 | 38 – 141 | 36 | 36 | 47 | 27 – 75 |
|  |  | UPAS |  |  |  |  |  |  |  |  |  |  |  |  |
|  |  | **ALL** | **35** | **35** | **75** | **112 – 276** | **64** | **39** | **56** | **30 – 104** | **74** | **39** | **38** | **26 – 61** |

**N_m_** stands for number of households; **N_hh_** stands for number of households; Grey-shaded area indicates no measurement available.

Figure S1. Quantile-Quantile Plot of Log-transformed PM_2.5_ measurements


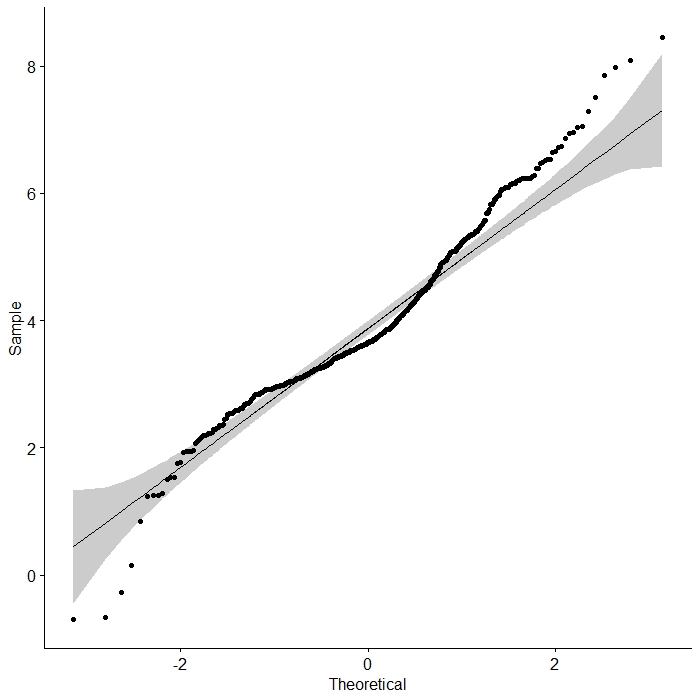

Supplement: Multimedia component 1 [file mmc1.docx]
